# A Comparative Study on the Microstructures, Mineral Content, and Mechanical Properties of Non-Avian Reptilian Eggshells

**DOI:** 10.3390/biology12050688

**Published:** 2023-05-07

**Authors:** Hsiao-Jou Wu, Yu-Chien Tseng, Shu-Han Tsao, Pei-Lin Chiang, Wei-Yu Tai, Hsin-I Hsieh, Hon-Tsen Yu, Jia-Yang Juang

**Affiliations:** 1 Department of Mechanical Engineering, National Taiwan University, Taipei 10617, Taiwan; r08b21004@ntu.edu.tw (H.-J.W.); r08522508@ntu.edu.tw (Y.-C.T.); heidi031158@gmail.com (S.-H.T.); pelin0817@gmail.com (P.-L.C.); 2 Department of Life Science, National Taiwan University, Taipei 10617, Taiwan; ayu@ntu.edu.tw; 3 Taipei Zoo, Taipei 11656, Taiwan; dwy85@zoo.gov.tw (W.-Y.T.); dwx03@zoo.gov.tw (H.-I.H.); 4 Degree Program of Genome and Systems Biology, National Taiwan University, Taipei 10617, Taiwan; 5 Program in Nanoengineering and Nanoscience, Graduate School of Advanced Technology, National Taiwan University, Taipei 10617, Taiwan

**Keywords:** reptilian eggshell, mechanical properties, microstructure, calcite, aragonite

## Abstract

**Simple Summary:**

Most previous studies on eggshell mechanics only focused on single species or taxa and often lacked a side-by-side comparison between eggshell microstructures, mineral content, and mechanical properties. However, those properties are essential to understanding amniotes’ ecology and evolution. Studies on non-avian reptilian eggshells are even more lacking than that of birds. In contrast to birds’ parental incubation, most reptilian eggs are hatched by environmental heat without parental care and are subjected to significant external disturbances. It is, however, still unclear whether reptilian eggshells have evolved different properties from birds due to their different incubation strategies. To fill this knowledge gap, we extend our previous work on bird eggs to cover various reptilian groups, including turtles, tortoises, crocodiles, and geckos, with 214 freshly laid eggs belonging to 16 species across three orders of Class Reptilia. This new result is compared to our previous work on birds and may shed new light on the correlation between eggshell properties and incubation strategies across a broader range of amniotic taxa (birds, crocodilians, turtles, tortoises, and geckos).

**Abstract:**

We analyze 214 freshly laid eggs belonging to 16 species across three orders of Class Reptilia. Using mechanical compression tests, we measure each egg’s absolute stiffness (*K*, unit: N m^−1^) and relative stiffness (*C* number). The effective Young’s modulus, *E*, was obtained by combining experimental and numerical methods. The mineral (CaCO_3_) content was measured by acid–base titration, the microstructures by scanning electron microscopy (SEM), and the crystallography by electron backscatter diffraction (EBSD). We find that the *C* number of reptilian eggs is, on average, higher than that of bird eggs, indicating that reptilian eggs are stiffer with respect to the egg mass than birds. However, Young’s moduli of the reptilian eggshells (32.85 ± 3.48 GPa) are similar to those of avian eggshells (32.07 ± 5.95 GPa), even though those eggshells have different crystal forms, microstructures, and crystallography. Titration measurement shows that the reptilian eggshells are highly mineralized (>89% for nine Testudines species and 96% for *Caiman crocodilus*). Comparing the species with aragonite and calcite crystals, we find that calcite shells, including those of the Kwangsi gecko (inner part) and spectacled caiman (outer part), generally have larger grains than the aragonite ones. However, the grain size is not correlated to the effective Young’s modulus. Also, as measured by the *C* number, the aragonite shells are, on average, stiffer than the calcite ones (except for the Kwangsi gecko), primarily due to their thicker shells.

## 1. Introduction

Amniotic eggs are a significant innovation that enable amniotes to colonize diverse terrestrial habitats [1] in addition to aquatic and humid environments. They provide the following essential functions for the embryos: (i) protection from damage and bacteria, (ii) control of the exchange of water and respiratory gases, and (iii) storage and provision of calcium for embryonic development [2]. Amniotic eggshells are traditionally categorized into three main types: hard (or rigid, used interchangeably in this paper), soft, and semi-rigid, according to the eggshell stiffness. Such a classification is somewhat subjective and may lead to errors in ancestral state reconstructions of eggshell microstructure [3]. However, avian eggshells are unequivocally rigid, and the stiffness of a given eggshell can be quantified by mechanical compression tests [4].

On the other hand, reptilian eggshells are not all rigid and can be divided into two groups according to their compositions: calcareous (including the “hard” and “semi-rigid” types) and leathery (“soft”) shells [5]. Rigid eggs include all birds, crocodilians, turtles, and geckos. Their shells mostly comprise a polycrystalline calcareous layer (CL) and are brittle and stiff [3,6,7,8]. Although rigid eggshells share similar layer structures, compositions, and brittleness, their microstructure and crystallography vary significantly across species [6,7,8]. For example, eggshells of Palaeognathae exhibit distinct microstructures and crystallography compared with those of Neognathae [6,9]; the growth direction of rigid gecko eggshells, e.g., *Gekko gecko*, is likely from the outer surface to the inner surface, which is opposite to that of archosaur eggshells (outward) [8]; other examples can be found in Legendre et al. [3].

Eggshell microstructures and mechanical properties are known to be constrained by specific reproductive strategies in birds [10,11,12] and non-avian reptiles (reptiles hereafter) [2,13] to achieve the functions mentioned above. Eggshell microstructures have been extensively studied in birds [6,7,9] and to a much less extent in reptiles such as geckos [8] and others [3]. Eggshell mechanical properties, such as stiffness and strength, have been studied for decades [12,14,15,16]. However, most of those studies were focused on a few common species, particularly chicken for the poultry industry. Thus, the relationship between microstructures and mechanical properties of eggshells over broad taxonomic scales remains largely unexplored. Chiang et al. studied the eggshell microstructures, mineral content, stiffness, and elastic moduli of 700 freshly-load eggs from 58 avian species. They found that the mineral content is positively correlated with Young’s modulus *E* (83.1% and 23.28 GPa for Zebra finch; 96.5% and 47.76 GPa for ostrich [7]), whereas the crystallography does not appear to correlate with Young’s modulus. However, similar studies for reptilian eggshells are still lacking.

Rigid eggshells are essential for avian reproduction as most birds use “contact incubation” and “egg turning” to provide a well-controlled condition for the developing embryos [17]. Thus, eggs must be stiff and strong enough to sustain the incubating birds’ weight and survive possible impacts between eggs or the surroundings. Eggshell stiffness (*K*, unit: N m^−1^) is conventionally measured by compression tests or finite element method (FEM) simulations [4,15] by compressing an eggshell along its long axis and can be expressed by $K=\left[2Et^{2}/\sqrt{3\left(1-\nu^{2}\right)}\right]\kappa$ , where *t* is the shell thickness, ν the Poisson’s ratio, and *κ* the local curvature at the pole. However, *K* is inadequate to compare eggshells’ *relative* stiffness among different species, as heavier eggs require higher stiffness to resist their weight or impacts from surrounding eggs. To overcome this, Juang et al. proposed a dimensionless metric, *C* number, to quantify eggs’ relative stiffness with respect to egg mass and shape, allowing for the comparison of eggshell stiffness across wide ranges of weight and shape [4,11,18]. Hung et al. analyzed 1350 species from 37 orders across the avian phylogeny [11]. They reaffirmed the general invariance of the eggshell relative stiffness observed in Ref. [4]. However, they also found that eggs in a more unsteady or enclosed nest have evolved higher stiffness, likely in response to higher collision risk in nests.

Such an invariance in the relative eggshell stiffness in birds is likely related to birds’ parental incubation. By contrast, most reptiles do not practice contact incubation and egg-turning. Instead, their eggs are hatched by environmental heat via diverse mechanisms, e.g., in soils, decaying vegetation, or exposure to the external environment [19]. Thus, most reptilian embryos develop under a microclimate that is much more diverse than the case of birds. Considering their distinct incubation strategies, it is intriguing to know how reptilian eggshells’ microstructures and mechanical properties differ from those of birds. Unfortunately, similar to the case of Young’s modulus, a comparative study on the eggshell stiffness of different reptilian groups has not been reported.

In this study, we aim to compare reptilian eggshells’ microstructures and mechanical properties, including turtles, tortoises, crocodiles, and geckos. Precisely, we measure the absolute and relative eggshell stiffness, *K* and *C*, using compression tests and obtain Young’s moduli, *E*, using the combined experimental and numerical method developed in [4]. Next, we measure the microstructures and crystallography using scanning electron microscopy (SEM) and electron backscatter diffraction (EBSD). Finally, we use acid–base titration to quantify mineral content.

## 2. Materials and Methods

In this study, we followed the methods of Juang et al. [4] to obtain the mechanical properties of eggshells. We followed the methods of Chiang et al. [7] for chemical contents and microstructures to compare the differences between bird and reptilian eggshells.

### 2.1. Egg Collection

**Figure 1:**
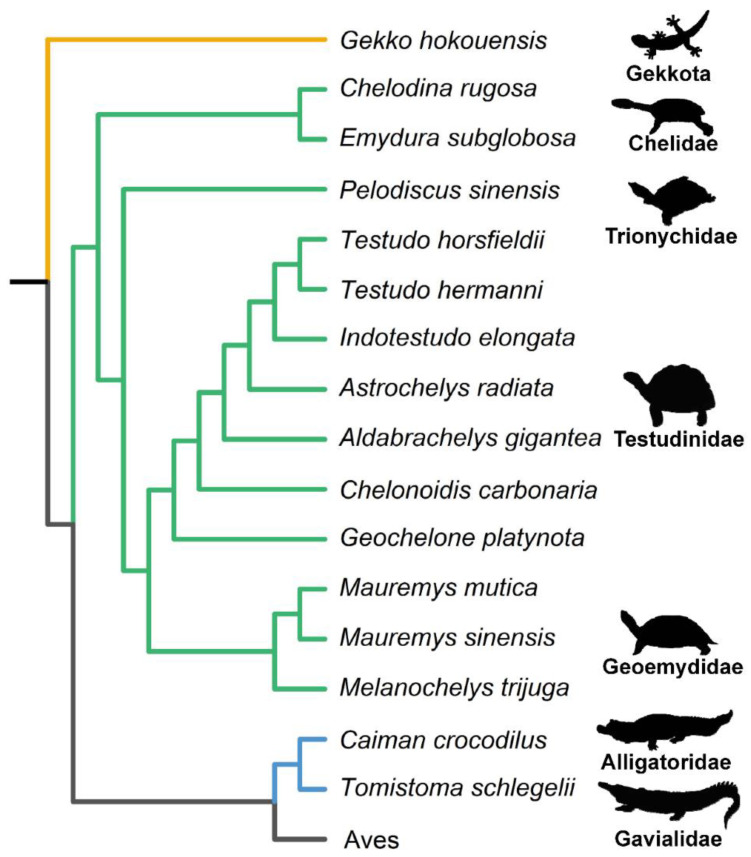
Phylogenetic relationships of reptiles based on molecular evidence. The phylogenetic tree was plotted with cladogram by package ggtree [28], and the colors of branches indicate three reptilian groups (yellow represents Gekkota of Squamata, green represents Testudines, and blue represents Crocodilia). The entire reptile relationship is based on the tree of Field et al. [29], and the phylogeny of Testudines is supplemented by Guillon et al. [30] and Le et al. [31]. The copyright of silhouettes is listed in Table S1.

**Figure 2:**
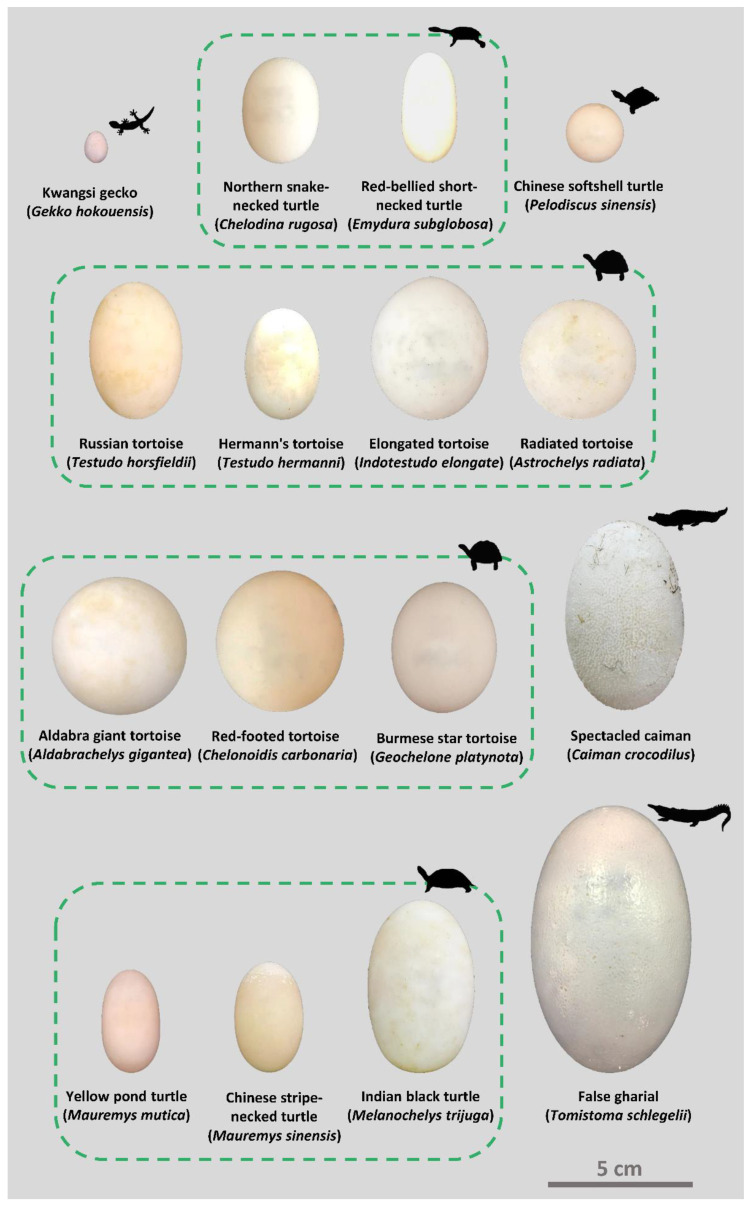
Egg specimens of sixteen species. The relative scale of specimens is based on actual sizes. Scale bar: 5 cm.

We analyzed 214 freshly laid eggs belonging to 16 species across three orders of Class Reptilia (Figure 1). Of all the egg specimens (Figure 2), 13 species belong to four families from Order Testudines (including turtles and tortoises), two species belong to two families from Order Crocodilia, and a species from Infraorder Gekkota in Order Squamata. Most egg samples were collected from Taipei Zoo, except eggs of spectacled caiman, which were acquired from Yizhu crocodile farm in Chiayi County, Taiwan. One of the major challenges of this study is egg collection. Therefore, we used all the egg samples we could acquire during this study over three years, from 2018 to 2020.

Our study did not compare eggs from wild individuals with captive individuals in zoos. However, the care of animals in Taipei Zoo is not like that of livestock animals but follows the best practice guidelines. In terms of environment, we mimicked the natural environment as much as possible so that animals could exhibit natural behaviors. The recipes were also as close as possible to their natural foods; if there were alternative foods, they would also have similar nutritional content. Thus, we may reasonably assume that the eggshell quality of the captive animals in zoos is similar to those in natural habitats.

A hen eggshell’s physical and chemical properties may change during the storage and feeding of the laying hens (and their ages). Much less is known for other bird species and (non-avian) reptiles. Unfortunately, the egg samples used in the present work were based on group breeding, and it is impossible to confirm the birth individual for every egg. Nevertheless, turtles are long-lived species and have decades-long breeding times. Furthermore, the egg samples used in this work were from healthy individuals with excellent reproductive status.

### 2.2. Mechanical Properties

In our previous studies on avian eggs [4], we defined a dimensionless number *C* (or *C* number) and introduced the method to estimate the effective Young’s modulus [7]. The *C* number quantifies the relative stiffness of an eggshell with respect to the egg size and can be used to compare the stiffness of eggs across wide ranges of weight and shape [4,11,18], and is defined as

(1) $$C\;\equiv \;\frac{K}{W}\frac{A^{2}}{B}$$

where *K* represents the (absolute) stiffness (unit: N m^−1^) measured by compression test (MTS Compression Machine Criterion Model E42), *W* is the egg weight (unit: N), and *A* and *B* are the width and length of the egg, respectively (unit: N m^−1^).

Using a computer simulation technique called finite element method simulation (FEM), we calculated the effective Young’s modulus (*E*_FEM_) for each sample after obtaining stiffness. In FEM, we assumed a uniform thickness and homogeneous eggshell; thus, *E*_FEM_ represents an overall rigidity of the shell, including the contribution of inorganic and organic constituents as well as pores and vesicles. However, due to the asymmetry of width in some Testudines species (Figure S1), we revised the simulation model to be closer to reality. In addition, the experimental data were corrected for machine compliance—if this is not done correctly, *E*_FEM_ will be underestimated. Table S2 provides data on egg mass, length, width, shell thickness, C number, and effective Young’s modulus. Unfortunately, flaws occurred for the eggs of false gharial (*Tomistoma schlegelii*) and Burmese star tortoise (*Geochelone platynota*) before the compression test; hence, we could not obtain the mechanical data for both.

### 2.3. Weight Percentage of CaCO_3_

Calcium carbonate (CaCO_3_) is the most abundant mineral content in eggshells [20]. Therefore, we quantified the weight percentage of CaCO_3_ as the main chemical content in eggshells by acid–base titration. At least three eggshell samples were picked for each species, but for some species with small eggs, multiple eggshells were merged into a single test as the test required a certain amount of material. The samples were ground into powder over a 75-μm filter. The first step was adding the eggshell powder to the prepared hydrochloric acid (HCl) solution. Next, stirring until the powder was completely dissolved. Then, HCl solution was titrated with sodium hydroxide (NaOH) solution. We calculated the weight percentage of CaCO_3_ when the mixed solution reached the equivalence point. The procedure was repeated at least three times for each reptilian species, and the average weight percentage of CaCO_3_ was obtained.

### 2.4. Scanning Electron Microscopy (SEM)

We used SEM (Phenom G2 Pro, Thermo Fisher Scientific Inc., Eindhoven, The Netherlands) to observe the reptilian eggshell transection microstructure to analyze the differences among reptilian groups. The SEM has a long-lifetime thermionic source electron beam with an accelerating voltage of 5.0 kV and a resolution of 25 nm. The electron-optical magnification can reach 45,000×.

The following are the fourteen reptilian species of SEM. Eleven species of Testudines: northern snake-necked turtle (*Chelodina rugosa*), red-bellied short-necked turtle (*Emydura subglobosa*), Chinese softshell turtle (*Pelodiscus sinensis*), Chinese stripe-necked turtle (*Mauremys sinensis*), yellow pond turtle (*Mauremys mutica*), Indian black turtle (*Melanochelys trijuga*), Aldabra giant tortoise (*Aldabrachelys gigantea*), radiated tortoise (*Astrochelys radiata*), red-footed tortoise (*Chelonoidis carbonaria*), elongated tortoise (*Indotestudo elongate*), and Russian tortoise (*Testudo horsfieldii*). Two species of Crocodilia: spectacled caiman (*Caiman crocodilus*) and false gharial (*Tomistoma schlegelii*). One species of Squamata: Kwangsi gecko (*Gekko hokouensis*).

### 2.5. X-ray Diffraction (XRD)

The eggshell crystal structure was analyzed using a Rigaku TTRAX 3 high-power X-ray diffractometer. The Cu Kα emission spectrum was used, which corresponded to an X-ray wavelength of 1.5406 Å, and the diffraction angle 2*θ* ranging from 20 to 60 degrees using a 0.02˚ step width. Comparing the XRD patterns with the database (Powder Diffraction File, JCPDS), we can determine the eggshell crystal structure (aragonite or calcite). We used Radiated tortoise, Aldabra giant tortoise, Elongated tortoise, and False gharialand eggshells as representatives.

### 2.6. Electron Backscatter Diffraction (EBSD)

The eggshell fragments were embedded in epoxy resin and processed in steps according to the EBSD requirement. First, each specimen was grinded sequentially by abrasive paper with seven different grain sizes and polished through 3 different sizes of alumina suspension by grinder-polisher (MetaServ 250, Buehler Ltd., Lake Bluff, Illinois, USA). The specimens were then coated with platinum for better conductivity. Next, the EBSD maps were obtained using the symmetry detector attached to the field emission scanning electron microscope (JeoL JSM-7800F Prime, JEOL Ltd., Tokyo, Japan) with the following setting: accelerating voltage 20.0 kV; 70 degrees tilting of specimens. According to previous studies, the crystal lattice of Testudines eggshells was set to aragonite [21,22], and the crystal lattice of gecko and crocodile eggshells was calcite [8,23]. Finally, the grain sizes of eggshell data were analyzed by the software AZtecCrystal (Oxford Instruments, Oxfordshire, UK). The species of EBSD samples were the same as those used in SEM.

## 3. Results and Discussion

### 3.1. Mechanical Properties

#### 3.1.1. Dimensionless Number *C* (*C* Number)

**Figure 3:**
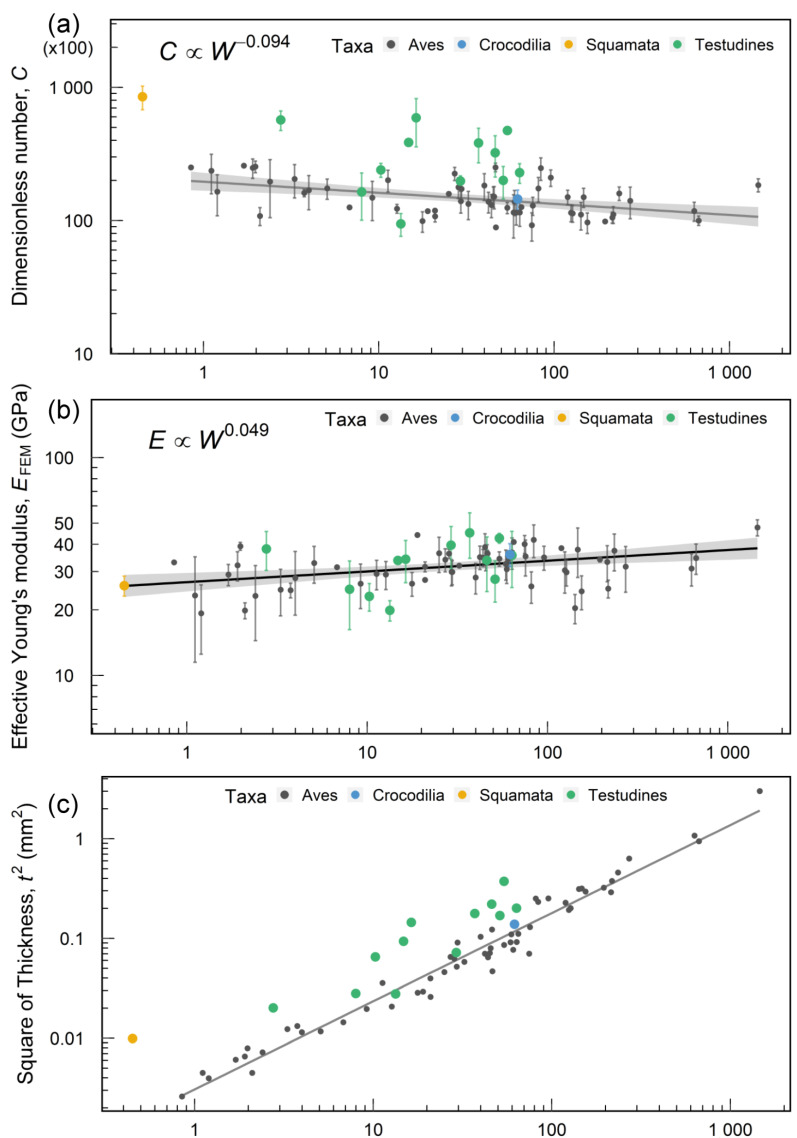
Mechanical properties vs. egg mass. (**a**) Dimensionless number, *C* (C number) of 77 species (point colors correspond to Figure 1). Per Symbol represents experimental measurements of one species (error bars represent standard deviation), the gray line represents the best-fit regression line of avian eggs, and the gray area represents the 95% confidence intervals. (**b**) Effective Young’s modulus, *E*_FEM_. The black line represents the best regression to all the data. (**c**) Square of eggshell thickness, *t^2^*. The gray line indicates the regression line of avian eggs.

The average *C* number of avian eggs is approximately 15,000, and it was found to be invariant in avian species [4]. Figure 3a shows the *C* number of the reptilian eggshell compared with avian eggs. Unlike the invariance in avian, the *C* number of reptilian eggs is less consistent. In comparing the *C* number, the standard deviation of birds is 4753.8, whereas that of reptiles is 16122.8. The maximum value is the egg of the Kwangsi gecko (*Gekko hokouensis*), which reached 84,897, and the minimum of reptilian eggs is 9442, belonging to the Chinese stripe-necked turtle (*Mauremys sinensis*).

For the species studied here, the *C* numbers of most reptilian eggs are higher than the average of avian eggs, indicating better ability to resist deformation in reptilian eggshells than in avian eggshells. Based on the relation of *C* number and eggshell thickness (*C* ∝ *Et*^2^/*W*), a thicker shell leads to a higher *C* number [4]. Figure 3c shows the comparisons among groups and the regression line calculated from avian egg mass and square of the thickness. The regression analysis shows that the eggshell thickness of Testudines and Kwangsi gecko (*Gekko hokouensis*) is significantly thicker than avian eggs with similar mass. Apart from the higher *C* values, the inter-species variation is higher in Testudines eggs. Unlike the contact incubation of birds [24], reptiles usually incubate their eggs in the environment. For example, crocodiles, turtles, and tortoises bury their eggs in the sand [19,25], and geckos lay eggs on the wall [26]. Perhaps due to unlimited incubation behaviors, the *C* number of reptiles displays a scattered distribution. The maximum *C* number of Testudines eggs belongs to the north snake-necked turtle (*Chelodina rugosa*), which lives in the tropic zone of Australia with distinct wet and dry seasons. North snake-necked turtles lay eggs in the hole, covered by shallow water; at that time, embryonic development would stop until the ground dried [27]. Therefore, North snake-necked turtles might have evolved stiffer eggshells to adapt to the changing environment. Twelve of the thirteen Testudines species studied here exhibit similar stiffer eggshells, compared to the avian case, which has an average *C* number of 15,000. Unlike Testudines species that bury their eggs in the sand, Kwangsi geckos lay their eggs in open environments without covering materials or protection. They thus may evolve stiffer eggshells, i.e., higher *C* value, to provide extra protection against external disturbances.

#### 3.1.2. Effective Young’s Modulus

Figure 3b compares effective Young’s modulus (EFEM) between reptilian and avian eggshells. The average (± standard deviation) *E*_FEM_ of avian eggshell is 32.07 ± 5.95 GPa, and the slope of the regression line is 0.047. After combining the data of reptilian eggs, the overall average of *E*_FEM_ is 32.22 ± 6.25 GPa, the slope of regression changes to 0.049, and residual sum of squares is 0.120, indicating that the effective Young’s modulus of all egg species is still invariant.

### 3.2. Weight Percentage of CaCO_3_

Even though the *E*_FEM_ is largely invariant, interspecific variants are still observable. In avian eggshells, the minimum *E*_FEM_ value belongs to society finch (*Lonchura striata domestica*), which is 19.28 GPa [7]; in reptiles, the minimum value is 19.90 GPa from Chinese stripe-necked turtle (*Mauremys sinensis*). The *E*_FEM_ 47.76 GPa of Ostrich (*Struthio camelus*) [7] and 45.14 GPa from radiated tortoise (*Astrochelys radiate*) represent the maximum of two groups. Previous studies showed that the mineral contents could impact the mechanical properties of biomaterials [7,32]. Calcium carbonate (CaCO_3_) is the major component in avian eggshells [33] and also reptilian eggshells [34]. Therefore, we obtained the weight percentage of CaCO_3_ of reptilian eggshells by acid–base titration.

**Figure 4:**
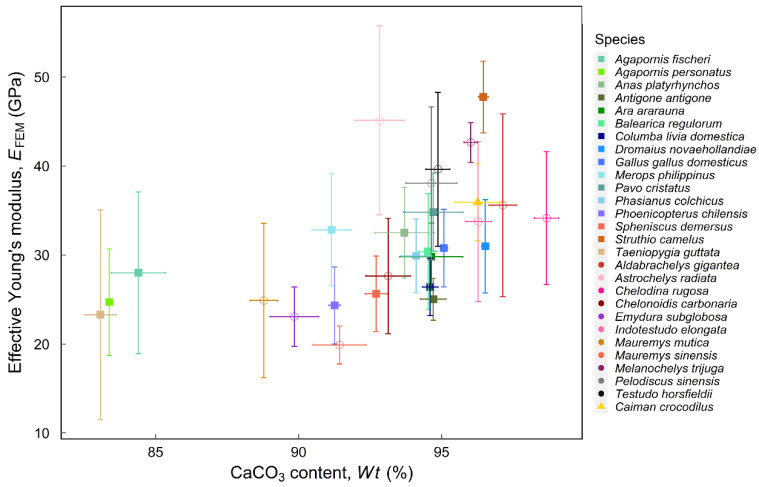
Young’s modulus vs. percentage of calcium carbonate in eggshells. Shapes of symbols indicate different groups: the square represents Aves, the circle represents Testudines species, and the triangle represents the species of crocodile. The detailed measurements are listed in Table S3.

Figure 4 shows the relationship between effective Young’s modulus and the weight percentage of CaCO_3_. The weight percentage of CaCO_3_ ranges from 83.07% to 97.14%, combining the avian data in Chiang et al. [7] (Table S3). As the figure shows, the weight percentage of CaCO_3_ is positively related to *E*_FEM_., albeit with considerable variations. However, there is no apparent correlation between CaCO_3_ percentage and *E*_FEM_, likely because the tested reptilian eggshells were all highly mineralized (>89%). Bone is another biomaterial mainly composed of calcium [35]. Studies on the long mammalian bone showed that the more the calcium, the higher the Young’s modulus [36]. The regression result is consistent with our data on eggshells.

### 3.3. X-ray Diffraction

**Figure 5:**
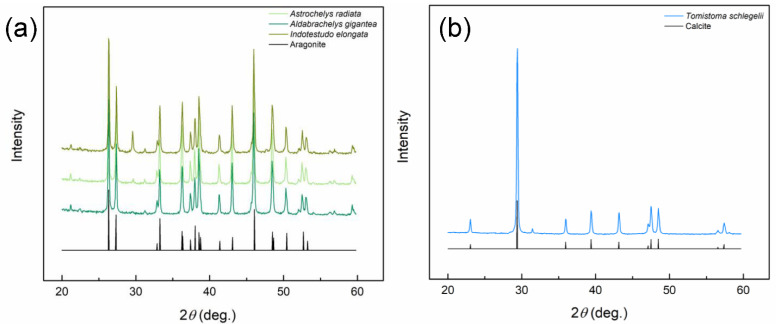
The X-ray diffraction (XRD) patterns of the eggshells of selected species. (**a**) Aragonite eggshells and (**b**) Calcite eggshell. The curves of Aragonite and Calcite represent the XRD of the single-crystal aragonite and calcite, respectively.

CaCO_3_ has three polymorphs in nature, including calcite, aragonite, and vaterite. Therefore, XRD analysis was performed on eggshell powder of several selected species to determine their crystal structures. As shown in the X-ray diffraction patterns (Figure 5), the peaks of the three turtle species aligned with single-crystal aragonite, whereas those of the crocodilian species aligned with single-crystal calcite. This result is consistent with that reported by Legendre et al. [3].

### 3.4. SEM Images of Eggshell

Although the reptilian and avian eggshells studied in this paper are both rigid (or hard) eggshells [3], there are various types within the structure [3]. We picked four species as representatives with specific characters. The rest of the species are listed in Figures S2–S5.

**Figure 6:**
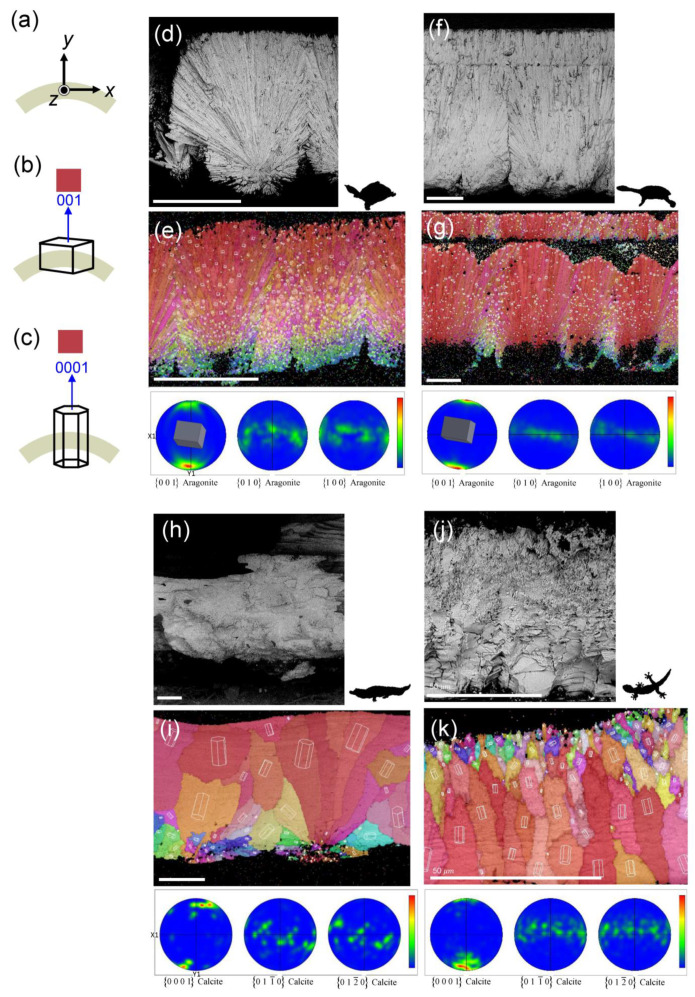
SEM and EBSD eggshell cross-section images of representative reptilian species. (**a**) The scanning direction of EBSD on a polished eggshell cross-section. The eggshell specimens under the Cartesian coordinate with the *y*-axis pointing to the thickness direction of the eggshell. The *x*-axis and *z*-axis are parallel to the eggshell surface. (**b**) The key of EBSD inverse pole figure map with aragonite form. When [001] direction of aragonite is parallel to the normal direction of the eggshell, the color in the inverse pole figure map appears red. (**c**) The key of EBSD inverse pole figure map with calcite form. When [0001] direction of calcite is parallel to the normal direction of the eggshell, the color in the inverse pole figure map appears red. (**d**,**f**,**h**,**j**) SEM image. (**e**,**g**,**i**,**k**) Inverse pole figure map of *y*-axis and pole figure in the XY-plane. (**d**,**e**) The eggshell of Chinese softshell turtle (*Pelodiscus sinensis*). The cuboid in the pole figure represents the preferred orientation of the grains in the eggshell. (**f**,**g**) The eggshell of north snake-necked turtle (*Chelodina rugosa*). (**h**,**i**) The eggshell of spectacled caiman (*Caiman crocodilus*). (**j**,**k**) The eggshell of Kwangsi gecko (*Gekko hokouensis*). Scale bar: 100 μm, except for gecko eggshell: 50 μm. A unit cell is displayed over each grain, showing the orientation for that grain in (**e**,**g**,**i**,**k**).

In Figure 6d, f is the sectioned eggshell of Testudines. Figure 6d shows the SEM photograph of the Chinese softshell turtle (*Pelodiscus sinensis*). It is the classical section-view of Testudines eggshells. The crystals of eggshells grow from the organic core, and the overall direction of growth is radiative, which is the same description in Schleich et al. [37]. Figure 6f shows the SEM photograph of the north snake-necked turtle (*Chelodina rugosa*). It contains a palisade layer except for organic cores and a radiative crystal layer. In the upper section of the SEM image, we can observe the structure of the crystal interrupt, which is shown in the image of Kusuda’s study as well [38].

Figure 6h displays the SEM image of the spectacled caiman (*Caiman crocodilus*) eggshell. It shows the regular horizontal layer, the typical crocodile eggshell structure [37]. Additionally, the tower structures occur on the outer side of the eggshell. They are mainly found in eggshells of the genus *Caiman* [39]. Figure 6j shows the SEM image of the Kwangsi gecko (*Gekko hokouensis*) eggshell. In the inner half of the eggshell, we could observe the jagged columnar layer. It occurs in other rigid eggshells of geckos, such as the ocelot gecko (*Paroedura picta*) and the tokay gecko (*Gekko gecko*) [8].

### 3.5. Crystallographic Analysis by EBSD

To determine whether crystal orientation or size might affect the mechanical properties of eggshells, we performed an EBSD analysis on fifteen reptilian species. The corresponding axes, denoting the directions on the eggshell, are shown in Figure 6a. The *x*- and *z*-axes are designated parallel to the shell surface, whereas the *y*-axis is in the thickness direction. Figure 6e,g,i,k displays EBSD images of four representative species with inverse pole figures (IPF) map along the *y*-axis (IPF-Y) and upper hemisphere pole figures in the *xy*-plane. The other species are shown in Figures S2–S5. In each figure of the IPF map, the outer layer is at the top, and the inner layer is at the bottom. Although reptilian eggshells are composed of calcium carbonate, there are two polymorphs of calcium carbonate. The eggshells of Testudines are comprised of aragonite [21,22], while calcite comprises eggshells of geckos and crocodiles [8,23]. Figure 6b,c represents the key to crystal orientations in IPF maps, displaying aragonite and calcite forms, respectively. The red refers to the [001] direction of the aragonite crystal parallel to the *y*-axis. Similarly, the red indicates y-axis is parallel to the calcite’s [0001] direction. Aragonite crystal belongs to the orthorhombic crystal system, shown as a cuboid, while calcite crystal belongs to the rhombohedral lattice system of the hexagonal crystal family, displayed as a hexagonal column.

#### 3.5.1. EBSD Maps

In an IPF map, an area of color represents a crystal grain; colors with close resemblance indicate that the growth direction of the grains is similar. With the key of Figure 6b,c, the red represents the [001] direction of aragonite and [0001] the direction of calcite. Besides, blue refers to $\left[10\bar{1}0\right]$ direction of calcite and [100] of aragonite, whereas green indicates the $\left[01\bar{1}0\right]$ direction of calcite and [010] direction of aragonite, respectively. The hemispheres of a pole figure represent the three orientations of crystal. The orientations from left to right for aragonite are $\left\{001\right\}$ , $\left\{010\right\}$ , and $\left\{100\right\}$ , whereas those for calcite are $\left\{0001\right\}$ , $\left\{01\bar{1}0\right\}$ , and $\left\{11\bar{2}0\right\}$ . If the color in the pole figure approaches red, it stands for the presence of the preferred orientation in the sample.

In the EBSD IPF maps of Testudines (Figure 6e,g), the aragonite grain size of the inner side is relatively small, and the orientation is more scattered than the outer. The IPF maps demonstrate the radiative growth direction, consistent with the SEM results. They also show that the grains have a strong [001] alignment in the main part of the shell, except for the inner surface, as shown by the reddish color and the aragonite cuboid overlaid on the map. Figure 6h shows some missing areas on the IPF map of the north snake-necked turtle (*Chelodina rugosa*), the inner palisade layer. The layer may be relatively fragile; therefore, it might be removed during the grinding process. Even though the inner palisade layer is missing, the IPF map still displays the interruption in the crystal disposition, and the second nucleation occurs at the outer palisade layer due to scattered grain orientations [38].

The calcite grains in the eggshells of geckoes and crocodiles appear larger and can display each grain. Figure 6i shows the IPF maps of spectacled caiman (*Caiman crocodilus*). The grain sizes of the inner eggshell are smaller and more scattered than the outside, the same as Testudines and avian eggshells [7,40]. The result of the pole figures shows one preferred grain orientation in caiman eggshell. In the IPF maps of the Kwangsi gecko (*Gekko hokouensis*), the differences in grain size between both sides reverse to the species mentioned above, as shown in Figure 6k. This observation is also displayed in the rigid eggshell of Choi’s study [8].

According to the color distribution of grains in IPF maps, we could infer the crystal growth direction in the eggshell [40]. In the primary growth of eggshells, crystals grow from the Testudines’ organic core from the mammillary core layer [38] or crocodiles’ nucleation center and basal knobs from the inner layer [41], expanding with the organic core as the center. The grains are smaller and have no preferred orientations of the inner eggshell, then getting larger with a fixed orientation, indicating the crystals grow outside [42,43]. On the contrary, the grain sizes are relatively smaller outside of the Kwangsi gecko eggshell, representing the origin of crystal growth at the outer [8].

If the crystals have consistent orientations, they will display similar colors on the IPF map, representing that the species’ eggshell has a texture [44,45]. For example, in EBSD images of Figure 6, the IPF maps are primarily red in the four representative species, and pole figures appear to prefer orientation in $\left\{001\right\}$ (aragonite) or $\left\{0001\right\}$ (calcite). This means that the [001] and [0001] directions of these crystals have a strong alignment along the *y*-axis, equal to the thickness direction, and represent the eggshell of four species have texture.

#### 3.5.2. Grain Size and Mechanical Properties

**Figure 7:**
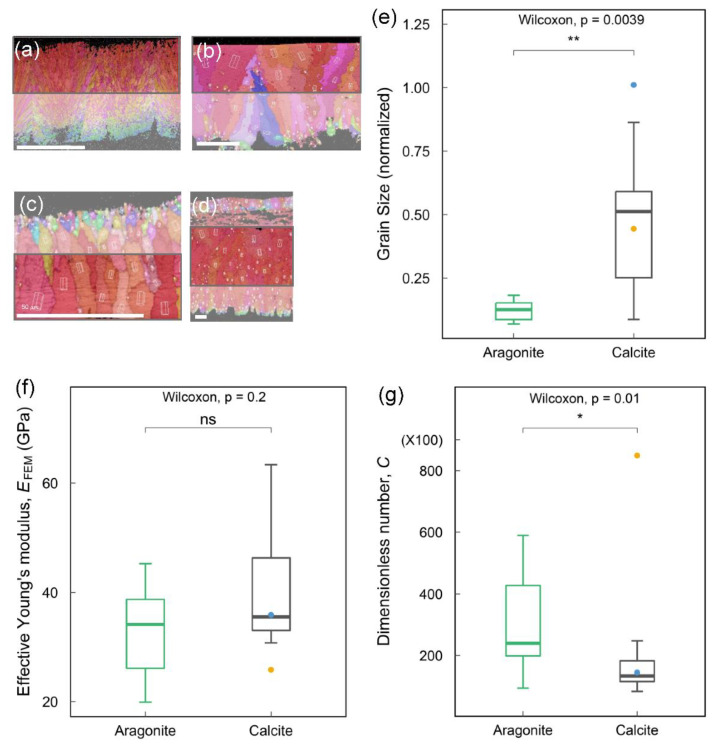
Comparisons of mechanical properties and grain size between two polymorphs of calcium carbonate. (**a**–**d**) Grain size calculating area framed by red lines. (**a**) Eggshell of Chinese softshell turtle (*Pelodiscus sinensis*) with a frame of the outer part. (**b**) Eggshell of mallard (*Anas platyrhynchos*) with a frame of the outer part. (**c**) Eggshell of Kwangsi gecko (*Gekko hokouensis*) with a frame of the inner part. (**d**) Eggshell of emu (*Dromaius novaehollandiae*) with a frame of the central part. (**e**–**g**) Comparisons of two polymorphs: aragonite and calcite. The data of aragonite included all species of Testudines. The data of calcite included all species of Aves but excluded crocodile species and gecko species; the two species are represented by points (blue is Spectacled caiman; yellow is Kwangsi gecko). (**e**) Comparison of normalized grain size. (**f**) Comparison of dimensionless number, *C.* (**g**) Comparison of effective Young’s modulus, *E*_FEM_. A unit cell is displayed over each grain showing the orientation for that grain in a−d. Statistical significance is indicated by asterisk * *p* < 0.05, ** *p* < 0.01.

In the research on cuckoos, the eggshells have higher strength due to smaller grain sizes and more scattered grain orientations [46]. Therefore, we quantified the results of EBSD to investigate the relation between grain size and effective Young’s modulus in reptilian eggshells. To normalize the grain size from each species, we used the equivalent grain perimeter divided by the shell thickness as the standard. The grains of inner eggshells in most species are too small and scattered to compare; we merely obtain the grains from upper shells (Figure 7a,b). Due to the growth direction of the gecko eggshell opposite to others, we obtained the inner part of the eggshell (Figure 7c). In avian eggshells, emu (*Dromaius novaehollandiae*) and northern cassowary (*Casuarius unappendiculatus*) have a reticular porous layer [47]. Therefore, the grains outside the special layer are relatively small, leading to errors in the entire data. In this case, we picked the center part of the shells (Figure 7d) and replaced the original value (*E*_FEM_: 30.97 GPa and 34.60 GPa) with the data removing the porous layer (*E*_FEM_: 68.4 GPa and 48.5 GPa). The wilcoxon signed-rank test was used in this statistic and analyzed by R 4.1.0 (R Foundation for Statistical Computing; Vienna, Austria) [48].

According to the crystal composition of eggshells, we separated aragonite and calcite to compare the standardization of both grain sizes. The dataset of aragonite includes species of Testudines (turtles and tortoises), whereas calcite data comprise all avian species but exclude the samples of crocodiles and geckos. The result shows that the normalized grain sizes of Testudines are significantly smaller than those of avian eggshells (Figure 7e). Next, we compared *E*_FEM_ and the C number based on crystal composition. Figure 7f shows that *E*_FEM_ exhibits no statistical significance between aragonite and calcite shells, suggesting that grain sizes do not affect the effective Young’s modulus in eggshells. Note that the effective Young’s modulus measures stiffness, not strength. The strength of the shells is not included in this paper and may be a topic for future work. As for the *C* number, the values of aragonite are significantly higher than those of calcite (Figure 7g), indicating that aragonite eggshells are stiffer than the calcite ones and deform less when subjected to the same load. Recall that the *C* number is proportional to *E* and *t*^2^, i.e., *C* ∝ *Et*^2^/*W*, and *E* has no significant difference in both groups. We conclude that the larger *C* values in aragonite eggshells are mainly due to their thicker shells, consistent with Figure 3c.

## 4. Conclusions

(1) No apparent trend was observed in the *C* number of (non-avian) reptilian eggshells compared to avian eggshells. However, the values of most Testudines and Kwangsi gecko (*Gekko hokouensis*) are higher than the average of avian eggshells for the species studied in this work.
(2) New data on the effective Young’s moduli (*E*_FEM_) of the eggshells of several reptilian species show that reptilian eggshells’ material rigidity is similar to that of avian eggshells, even though those eggshells have different crystal forms, crystallographic, and microstructural characteristics. The overall average of *E*_FEM_ is 32.22 ± 6.25 GPa.
(3) Our titration result shows that the weight percentage of CaCO_3_ is positively correlated to the effective Young’s modulus.
(4) The EBSD analysis revealed that all the reptilian eggshells have textures exhibiting a preferred grain orientation during growth. By contrast, eggshells of some avian species have weak or no texture (weak or no vertical *c*-axis alignment), as shown in our previous work [7] and Choi et al. [6].
(5) Comparing the species with aragonite and calcite crystals, we found that calcite shells, including those of the Kwangsi gecko (inner part) and spectacled caiman (outer part), generally have larger grains than the aragonite ones. However, the grain size was not correlated to the effective Young’s modulus. Also, as measured by the *C* number, the aragonite shells are, on average, stiffer than the calcite ones (except for the Kwangsi gecko), primarily due to their thicker shells.
(6) Our conclusions were mainly based on Testudines with a comparison with representative samples from Crocodiles and Squamata. Future studies may consider including more species from orders other than Testudines.

## Data Availability

The data presented in this study are available on request from the corresponding author.

## Supplementary Materials

The following are available online at https://www.mdpi.com/2079-7737/12/5/688/s1, Figure S1: Top view of egg length direction; Figure S2: SEM and EBSD images of Chelidae species; Figure S3: SEM and EBSD images of Testudinidae species; Figure S4: SEM and EBSD images of Geoemydidae species; Figure S5: SEM and EBSD images of Gavialidae species; Table S1: copyright information about reptiles images; Table S2: dataset of eggs, including *C* numbers; and Table S3: dataset of eggs, including mineral content.
