# Supplementary material for: A Comparative Study on the Microstructures, Mineral Content, and Mechanical Properties of Non-Avian Reptilian Eggshells
Source: Biology (Basel). 2023 May 7;12(5):688. doi: 10.3390/biology12050688 (PMC10215611; doi:10.3390/biology12050688)
Supplement: Supplementary file 1 [file biology-12-00688-s001.zip › Figure of reptile eggs supplementary.pdf]

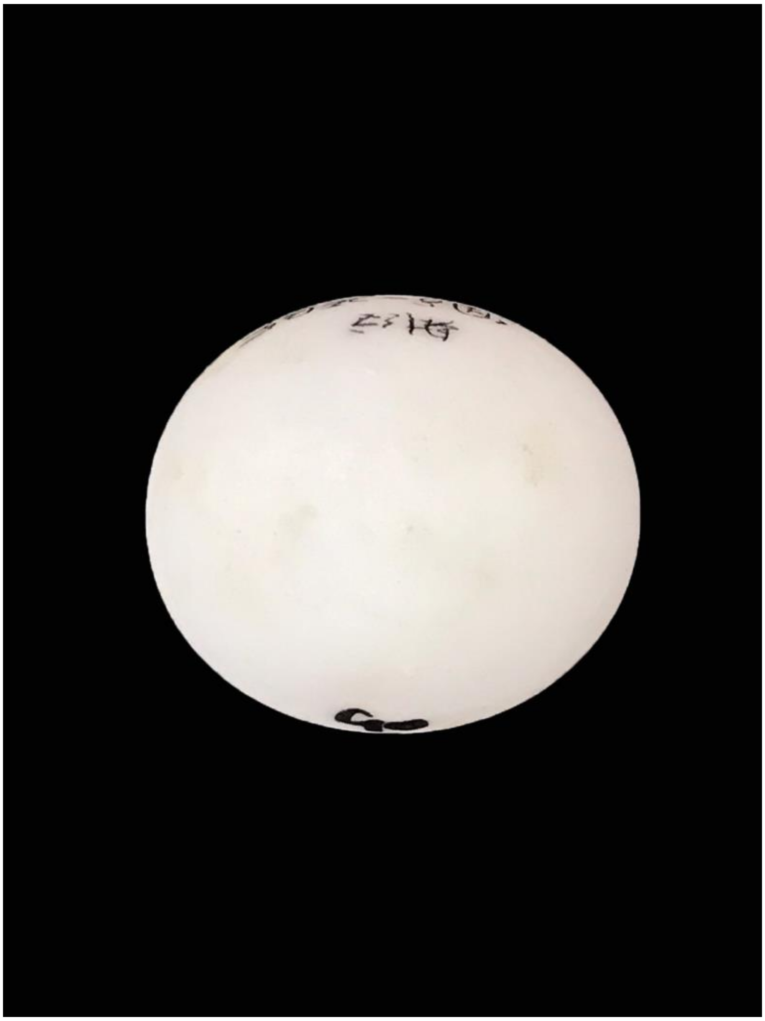

Fig. S1. Top view of egg length direction. The specimen belongs to radiated tortoise (*Astrochelys radiata*).

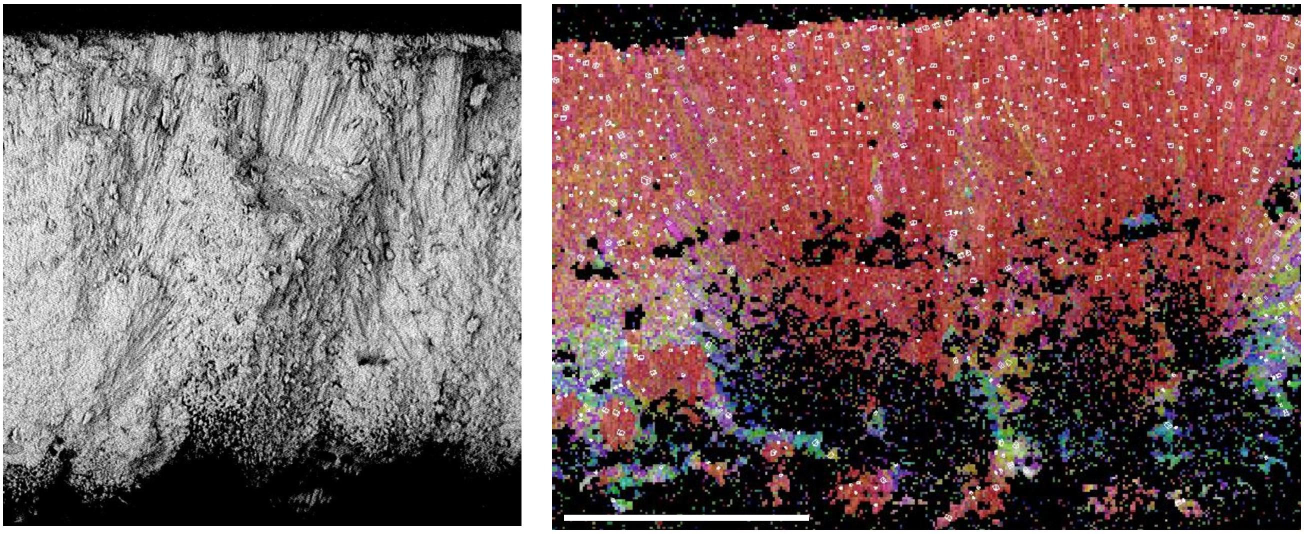

Red-bellied short-necked turtle (*Emydura subglobosa*)

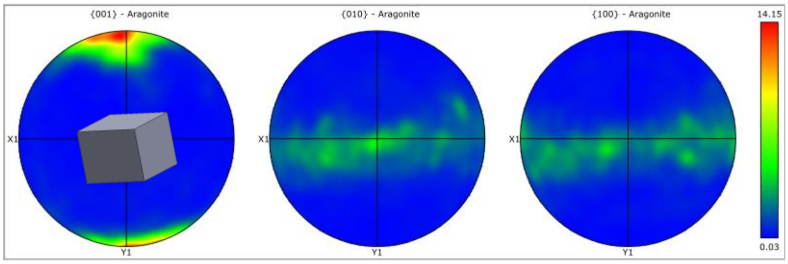

Fig. S2. SEM and EBSD Images of Chelidae species. Red-bellied short-necked turtle (*Emydura subglobosa*). Scale bar: 100 µm.

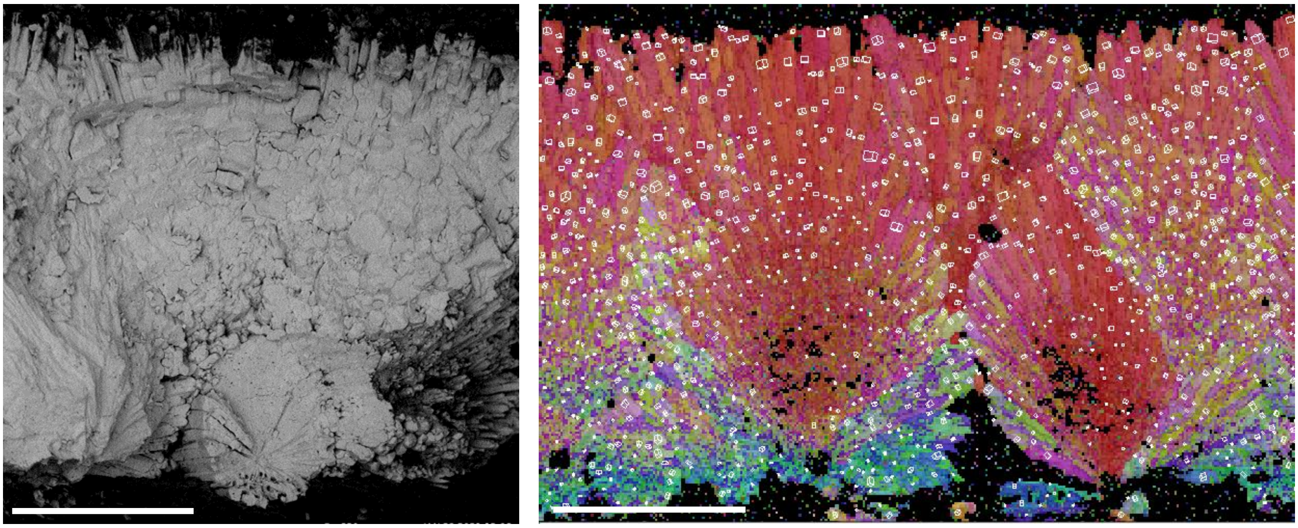

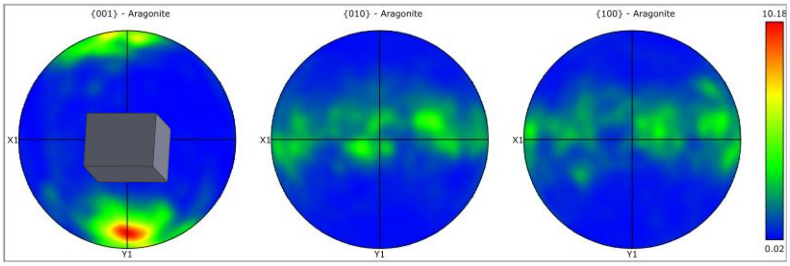

A

Russian tortoise

(*Testudo horsfieldii*)

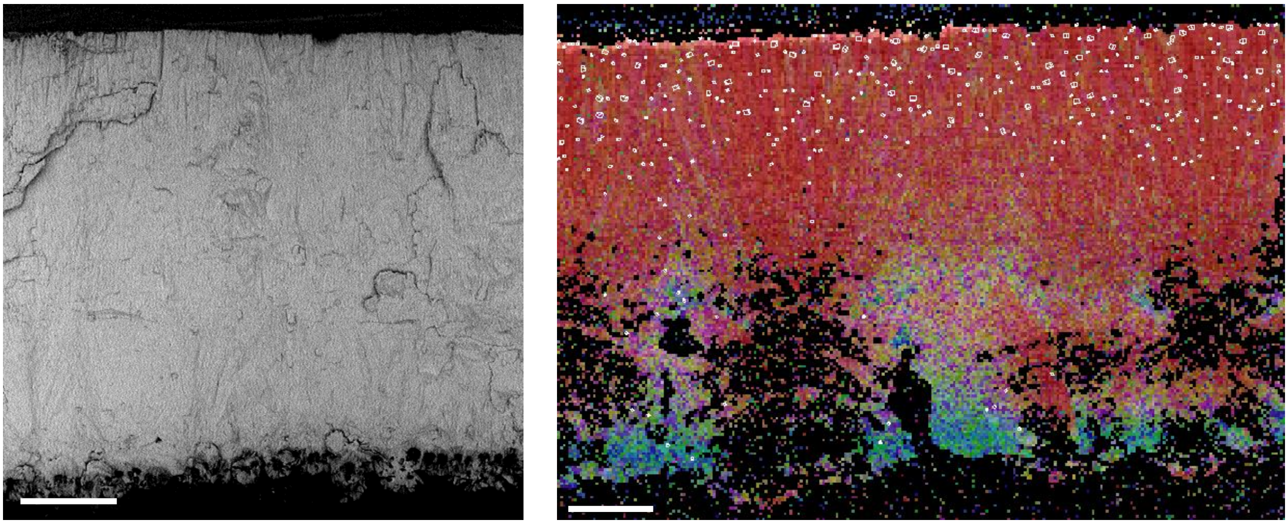

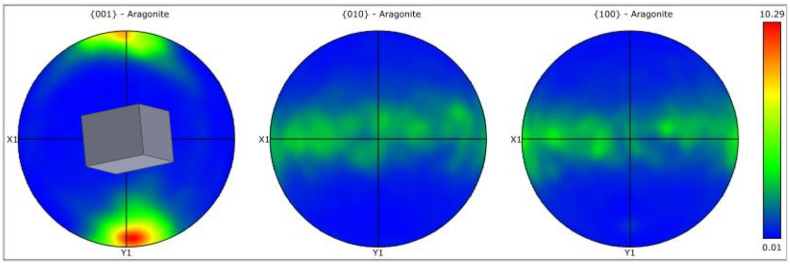

B

Elongated tortoise

(*Indotestudo elongate*)

Fig. S3. SEM and EBSD Images of Testudinidae species. (A) Russian tortoise (*Testudo horsfieldii*). (B) Elongated tortoise (*Indotestudo elongate*). Scale bar: 100 µm.

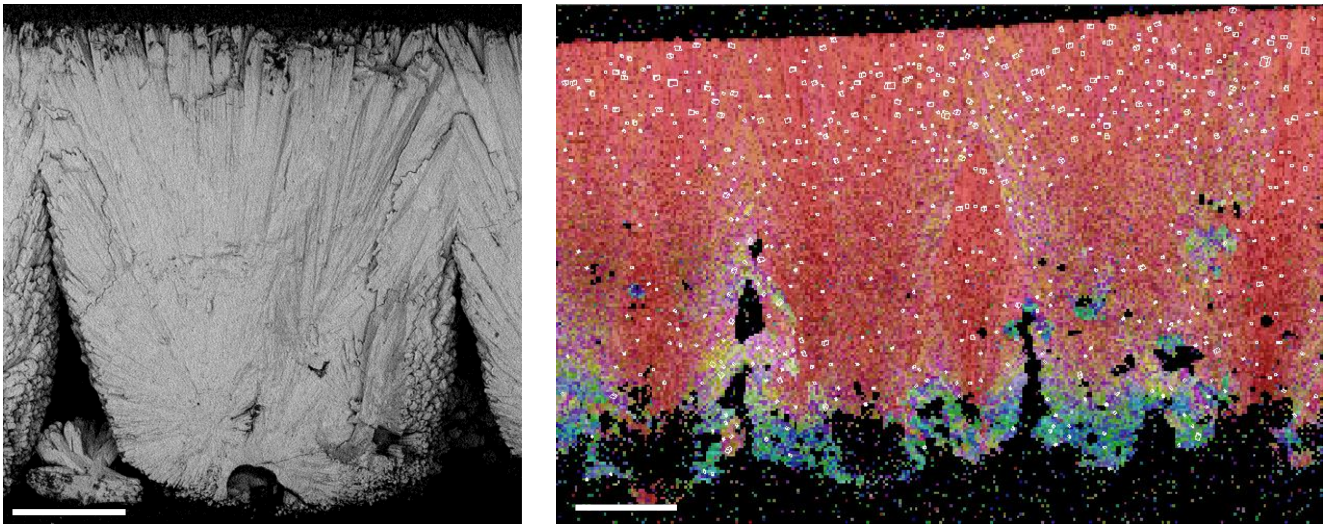

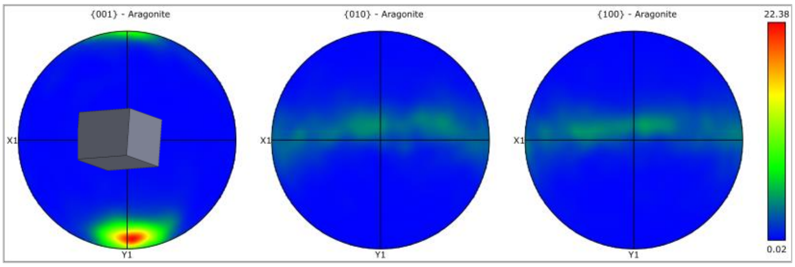

C

Radiated tortoise
(*Astrochelys radiata*)

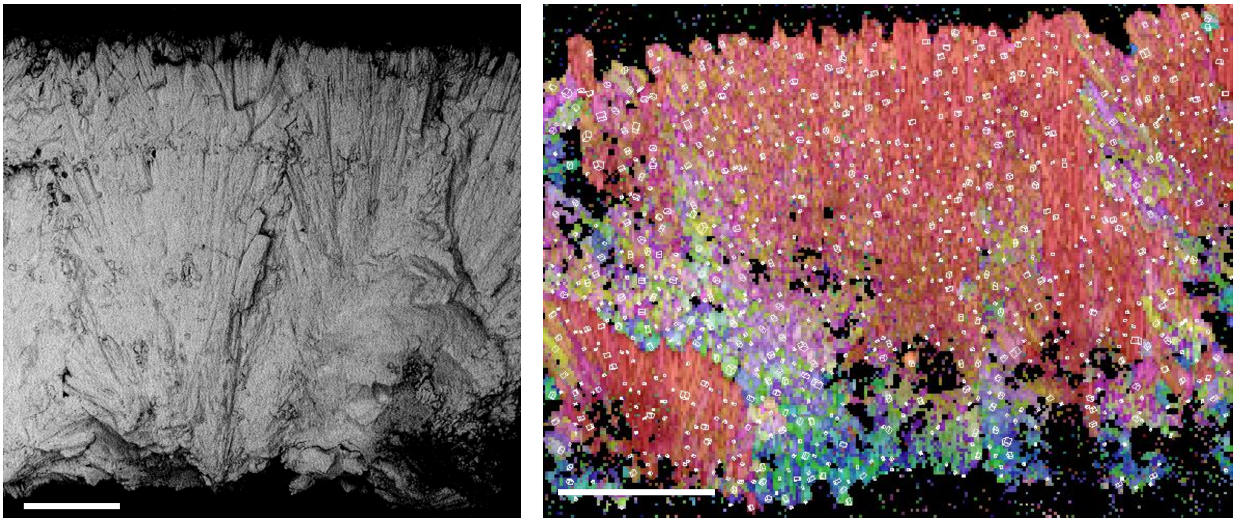

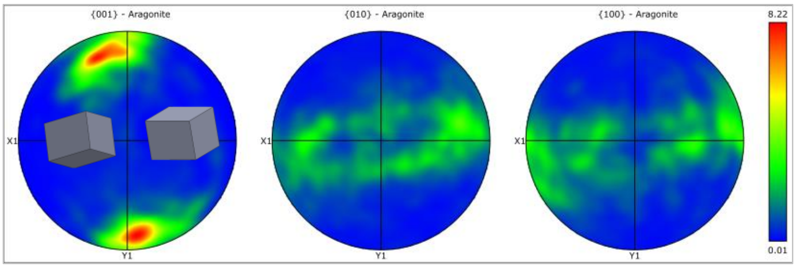

D

Aldabra giant tortoise
(*Aldabrachelys gigantea*)

Fig. S3. SEM and EBSD Images of Testudinidae species (Continued). (C) Radiated tortoise (*Astrochelys radiata*). (D) Aldabra giant tortoise (*Aldabrachelys gigantea*). Scale bar: 100 µm.

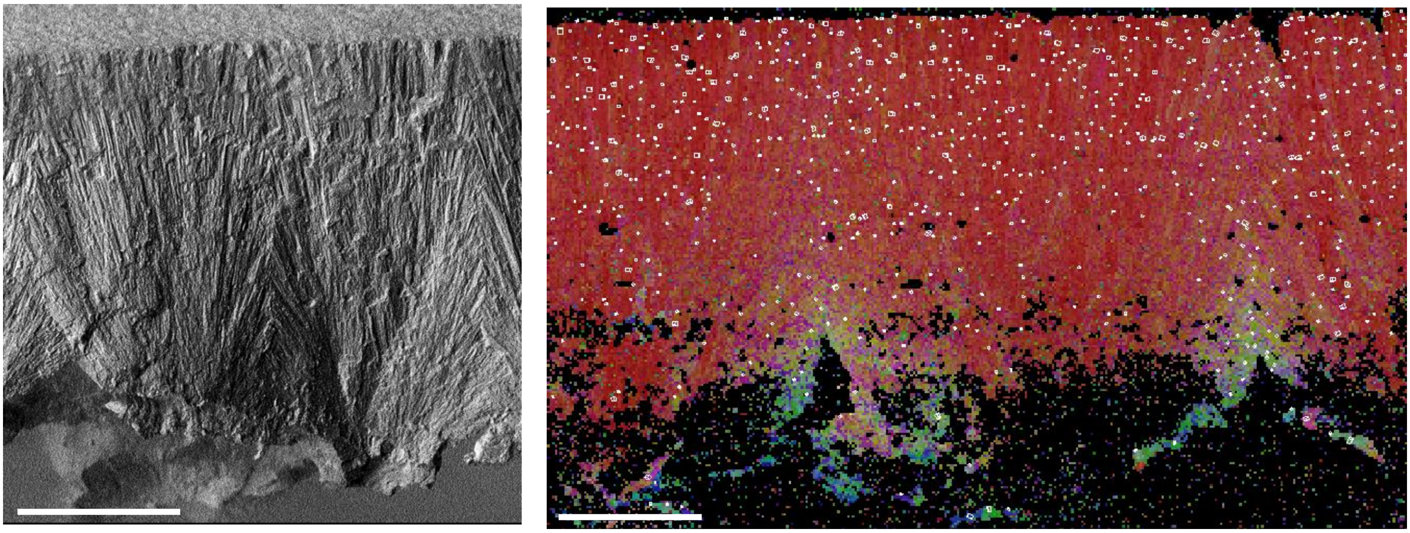

E

Red-footed tortoise
(*Chelonoidis carbonaria*)

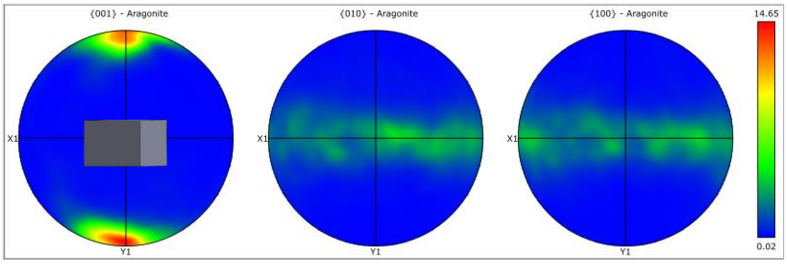

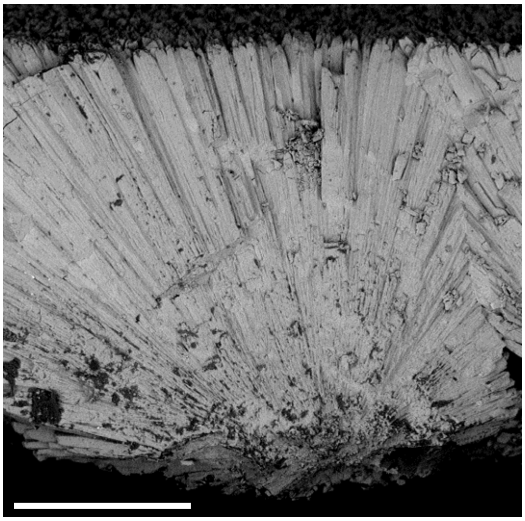

F

Burmese star tortoise
(*Geochelone platynota*)

Fig. S3. SEM and EBSD Images of Testudinidae species (Continued) . (E) Red-footed tortoise (*Chelonoidis carbonaria*). (F) Only SEM image in Burmese star tortoise (*Geochelone platynota*). Scale bar: 100 µm.

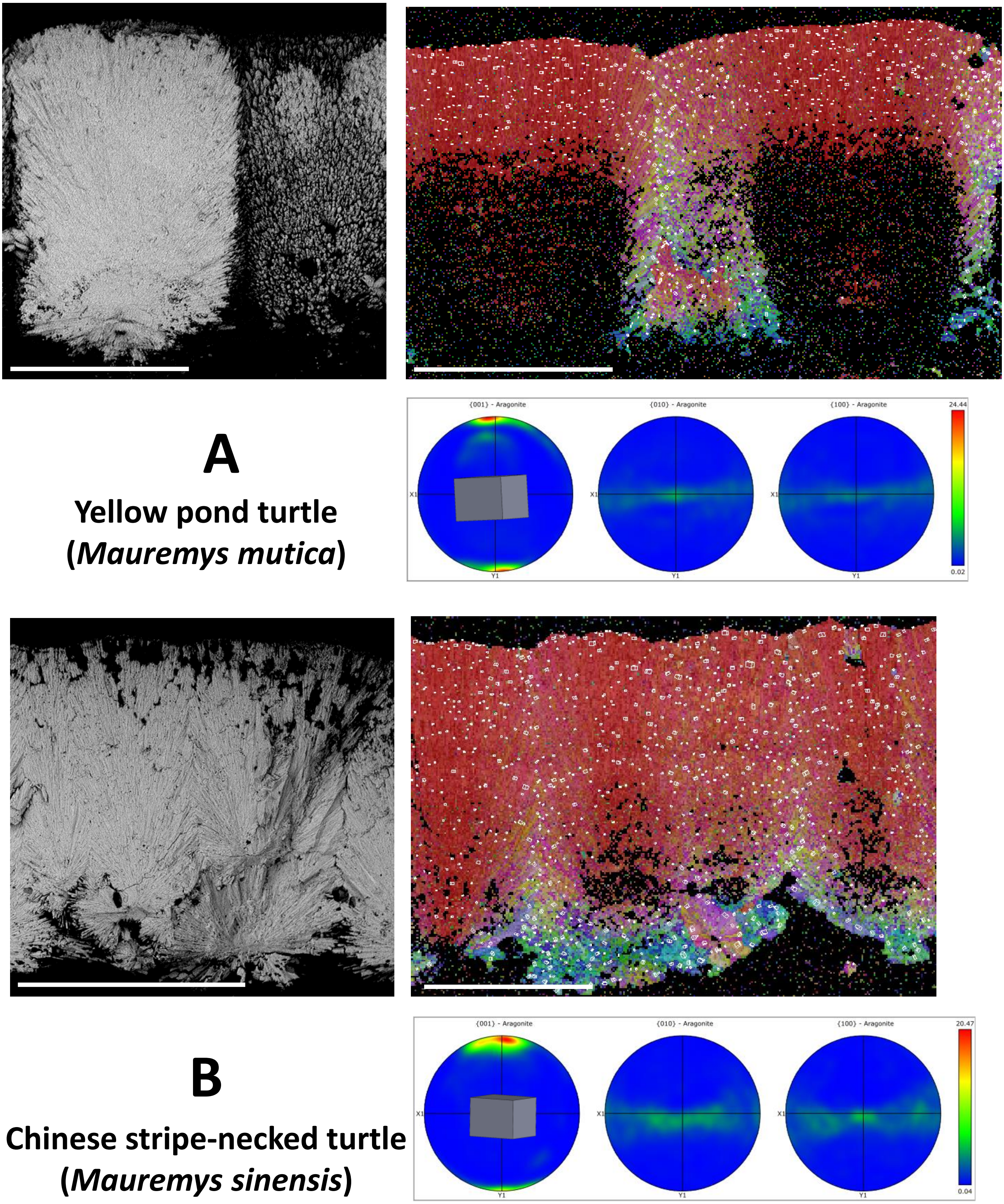

Fig. S4. SEM and EBSD Images of Geoemydidae species. (A) Yellow pond turtle (*Mauremys mutica*). (B) Chinese stripe-necked turtle (*Mauremys sinensis*). Scale bar: 100 μm.

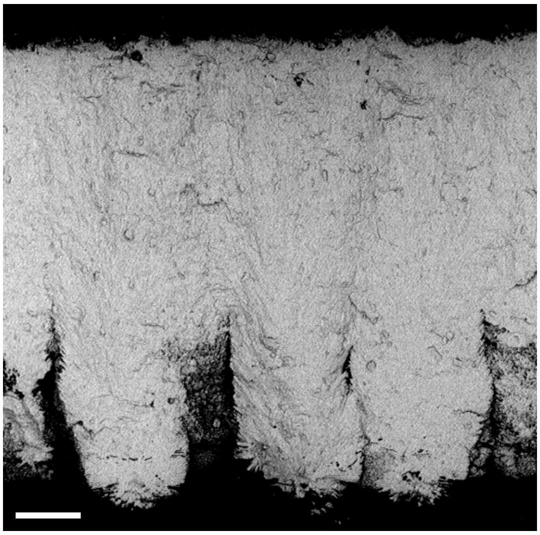

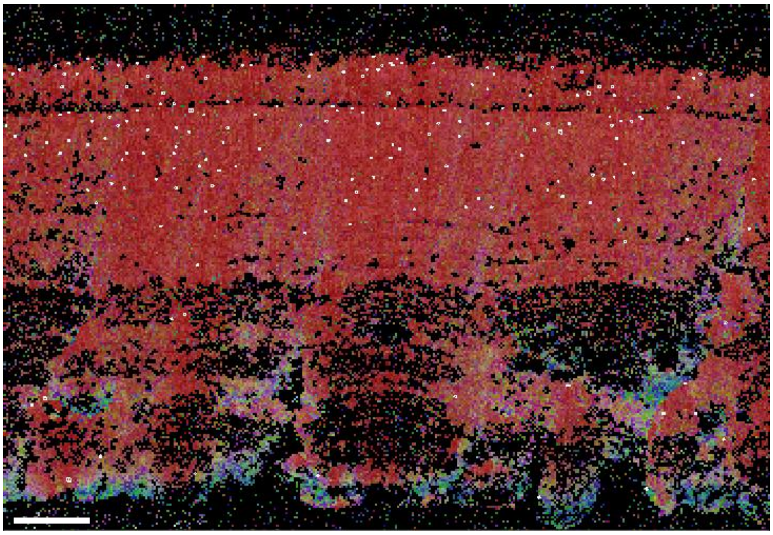

C

Indian black turtle
(*Melanochelys trijuga*)

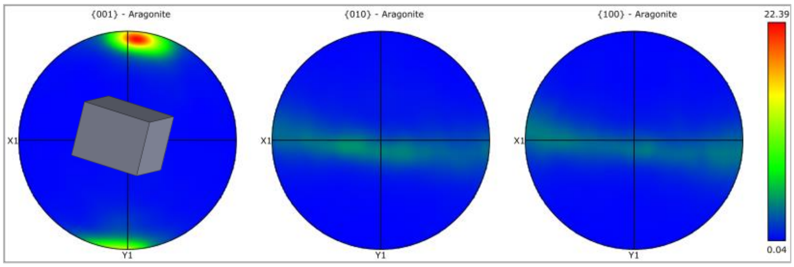

Fig. S4. SEM and EBSD Images of Geoemydidae species (Continued) .
(C) Indian black turtle (*Melanochelys trijuga*). Scale bar: 100 µm.

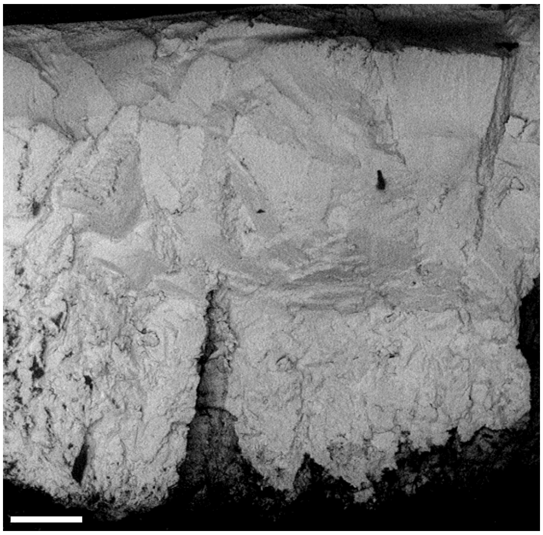

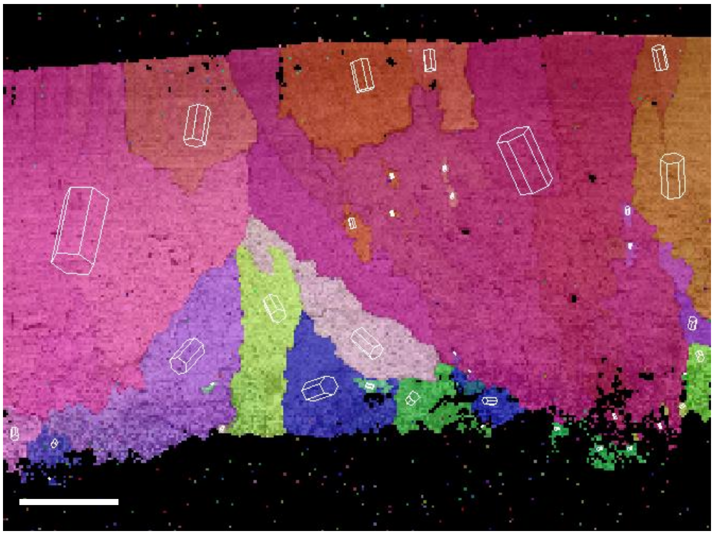

False gharial
(*Tomistoma schlegelii*)

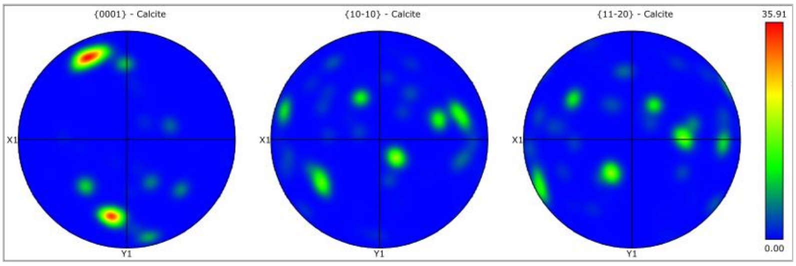

Fig. S5. SEM and EBSD Images of Gavialidae species. False gharial (*Tomistoma schlegelii*). Scale bar: 100 µm.
